# Critical risk identification in construction projects using 2D and 3D risk matrices

**DOI:** 10.1371/journal.pone.0344476

**Published:** 2026-03-05

**Authors:** Shamal Ali Othman, Dalshad Kakasor Ismael Jaff, Ahmet Öztaş

**Affiliations:** 1 Department of Civil Engineering, College of Engineering, Salahaddin University-Erbil, Erbil, Kurdistan Region, Iraq; 2 Department of Civil Engineering, Epoka University, Tirana, Albania; Kwame Nkrumah University of Science and Technology, GHANA

## Abstract

Construction projects are exposed to numerous risks that may adversely affect project cost and duration. The purpose of this study is to identify and prioritize key construction risks by combining a Work Breakdown Structure (WBS) with a Risk Breakdown Structure (RBS) and analyzing these risks using two-dimensional (2D) and three-dimensional (3D) risk matrices. In total, 63 risk factors were identified and categorized into five main areas of the work breakdown structure: earthwork, concrete, finishing, mechanical, and electrical. The questionnaire was designed based on each risk category and then sent to engineers, who were asked to estimate the risk probability and its impact. In total, 378 engineers responded. The respondents data were analyzed by using the risk matrix method. Two separate 3D risk matrices were made: one was based on multiplying all three data points, and the other was based on combining both impacts. The results clearly show that the 3D matrix, which combines cost and time impact, yields more accurate results than the matrix that multiplies cost impact and time impact.

## Introduction

Risk and uncertainty can be considered as crucial elements in construction projects that need effective management to achieve project goals. Construction projects involve various activities that can be categorized under different work structures. Even though every task is meticulously planned and managed, it is crucial to accurately identify and evaluate the risks involved in each activity. Risk might pose as a threat or a favorable opportunity. Threats should be controlled and planned by utilizing a risk management framework, which is a systematic process that begins with identifying risks, then analyzing them, and then implementing mitigation solutions.

The majority of studies have focused on different risk breakdown structures for identifying risks. However, construction projects continue to demand more thorough methods for recording risks. A risk breakdown structure (RBS) is considered the best practice for defining and grouping risks into different levels [1]. Hillson [1] defined RBS as “A source-oriented grouping of project risks that organizes and defines the total risk exposure of the project. Each descending level represents an increasingly detailed definition of sources of risk to the project.” A more effective solution to the structural challenges in risk management is to implement a fully hierarchical approach similar to that used in a Work Breakdown Structure (WBS). WBS is a scope management tool developed by the United States Department of Defense. It breaks down intricate and methodical tasks into more manageable, quantifiable work bundles [2]. Construction projects face various kinds of risks, and each activity may have specific risk factors that can increase costs and extend activity duration. It is crucial to identify risks while considering the WBS. For identifying the risk in construction projects, this study tries to develop a risk breakdown structure in conjunction with a work breakdown structure specifically for construction projects.

Following risk identification, risk analysis is conducted to evaluate the likelihood and consequences of identified risks [3]. Risk analysis can be performed using qualitative or quantitative approaches, depending on data availability and project context [4]. In construction management, risk analysis is closely related to two fundamental questions: How long will the project take? and How much will it cost? [4]. Qualitative risk analysis commonly relies on risk scores to prioritize risks based on their probability and impact. While many studies consider impact as a single dimension, construction risks frequently affect both cost and time. To address this, some researchers have proposed three-dimensional (3D) risk matrices. Yet, a significant gap remains in the mathematical formulation of these 3D models. Existing studies rarely question whether risk dimensions should be multiplied (Multiplicative Model: P * Ic * It or combined (Combinatorial Model: (P * [Ic + It]).

Therefore, this study goes beyond simple identification. The main objectives are: (1) to utilize an integrated RBS-WBS structure to systematically identify risks, and (2) to empirically compare two distinct mathematical risk scoring models (Multiplicative vs. Combinatorial) within a 3D matrix. This comparison seeks to determine which mathematical formulation provides a more accurate representation of risk criticality in construction projects.

## Literature review

Risk management is a fundamental component of successful construction project delivery. The construction project's planning begins with the preparation of a WBS. This process involves dividing the project into smaller, more manageable components [5]. However, recent trends indicate that managing scope alone is insufficient; risk must be integrated directly into these structures. This section reviews the integration of breakdown structures, the evolution from 2D to 3D assessment, and limitations of 2D risk matrices and the emergence of 3D models.

### Integration of WBS and RBS

RBS and WBS were integrated in different studies to identify risks in construction projects and define risk factors more effectively. Agistin, et al. [6] developed a risk-based, standardized WBS in construction projects in Indonesia to improve time performance. Li, et al. [7] integrated RBS and WBS for identifying risk factors in bridge projects. Supriadi, et al. [8] developed a standard WBS and investigated the potential risks connected with project implementation. They concentrated on looking at the risk issues associated with the normal WBS activities from the perspective of the contractors.

The WBS and RBS integration helps project managers to identify risk factors more easily and more accurately. Such integration also improves the recognition of risks, as it allows the decision-makers to make more objective and efficient management decisions and to effectively distribute resources in a multi-project environment [2,9–11]. Recent research supports this opinion that this integration is not only helpful but essential. Atmaja, et al. [12] argued that as long as there is no standard WBS-RBS framework, risk identification will be fragmented and will not identify the fact where particular deliverables are affected by risks. As well as Hartanti [13] applied a risk-based WBS to minimize potential risks. Othman, et al. [14] concluded that RBS integrated with WBS results in a more realistic, structured, and reliable risk assessment. Finlay, it can be concluded that the WBS and RBS can be used to create a matrix structure that can help the project team in managing the risks at a level of detail that is appropriate for the particular business environment [15].

### Risk matrix as a tool for risk assessment

Risk matrices are among the most widely used tools for risk assessment due to their simplicity and practicality [16]. A risk matrix typically classifies risks based on probability and impact, enabling risks to be categorized as low, medium, or high [17]. Anthony Cox Jr [16] described the risk matrix as a chart with likelihood categories on one axis and impact categories on the other. Various matrix sizes, such as 3 × 3, 4 × 4, and 5 × 5, are commonly applied in practice [16].

The traditional risk matrices have risk scores that are calculated by multiplying the probability of occurrence by the impact of the potential loss [18,19]. Murray, et al. [20] introduced a generic risk matrix for contingency planning with limited requirements in terms of resources, while Luo, et al. [21] integrated risk matrices with other analytical methods for risk assessment in complex engineering projects. From these studies, it is clear that the use of risk matrices are widely used in various fields. However, most applications use 2D formulations, which take impact into account as one total measure.

### Limitations of 2D risk matrices and emergence of 3D models

Despite their popularity, however, 2D risk matrices have certain notable limitations, especially in projects where risks have simultaneous impacts on different performance dimensions. In construction projects, the 2D risk model remains the most commonly used approach [19,22,23]. However, the consideration of impact as a single dimension may conceal the different impacts of risks on cost and time [24].

In order to overcome these limitations, 3D risk assessment models have been proposed. The University of Melbourne's Risk Management Office developed a 3D risk model, which includes additional dimensions of impact, although it does have some challenges in graphical representation, and it is difficult to obtain a reliable representation of the risk [22]. Korobeynikov [25] showed how 3D risk matrices can be effective in dealing with complex critical risks, especially in resilience and information security fields. These models combine the probability with multiple dimensions of impact, resulting in a more comprehensive model of risk.

While existing studies recognized the conceptual advantages of 3D risk matrices, most focus on model development or single-model applications. Limited research has empirically compared alternative 3D risk matrix formulations or examined how different formulations influence risk ranking consistency and prioritization in construction projects. This gap highlights the need for systematic evaluation of competing 3D risk scoring approaches within a unified risk identification framework.

## Methodology

### Ethics

This research was undertaken as a component of a PhD dissertation. The waiver was granted because the study consisted of an anonymous questionnaire survey regarding professional opinions, was devoid of personal identification or sensitive data, and did not involve clinical interventions or experimentation on human subjects. Participation was voluntary, and submission of the completed questionnaire was interpreted as implied consent.

### Risk identification

**Fig 1:**
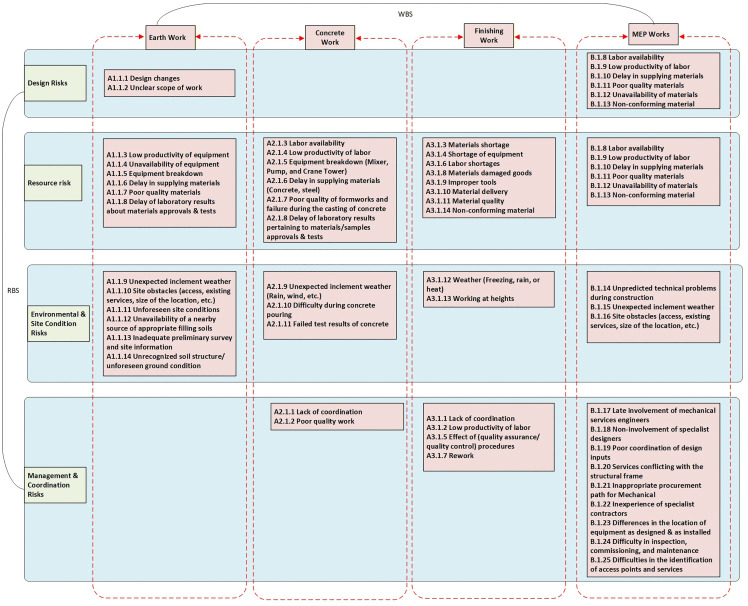
RBS and WBS Integration.

The main objective of this study is to develop a risk matrix that considers the risk factors that may occur during different types of work in projects. The risk factor was identified based on the integration of RBS with WBS. The main tasks in WBS for construction projects include earthwork, concrete work, finishing work, electrical work, and mechanical and plumbing work. RBS was established by reviewing various studies related to construction projects [6–8,26–29]. The main risk categories in RBS include design risks, resource risks, environmental and site condition risks, and management and coordination risks. There is a total of 63 risk factors categorized under RBS and WBS, as shown in Fig 1. Experts can estimate the probability and impact of risks through questionnaires [30].

### Questionnaire design

A questionnaire was designed to determine the risk probability and impact. The questionnaire consists of two main parts. Part 1 includes general and qualitative questions included general questions about the respondents profile (experience, education, job title). These questions yielded qualitative (nominal) data used to verify the expertise of the participants. The second part is quantitative, that contained the specific risk factors that listed in Fig 1. Respondents were asked to rate the probability, cost impact, and time impact on a numerical scale. These questions yielded quantitative data necessary for the matrix calculations. The 5-point Likert scale was utilized to quantify the probability of occurrence for each risk, as well as its impact on costs and time. The risk factors included in the questionnaire were organized under each WBS category, which includes fourteen risk factors associated with earthwork, eleven risk factors related to concrete work, fourteen risk factors for finishing works, and twenty-five risk factors related to mechanical, electrical, and plumbing works. The data were collected by distributing a questionnaire link to engineers working in different sectors of the construction industry, who were asked to complete the questionnaire form.

### Determining the sample size

Sample size determination for multi-item Likert-scale questionnaires requires approaches that go beyond conventional heuristics, which have been shown to frequently underestimate the required number of responses and lead to under powered empirical results [31]. In response to these limitations, this study adopted the method proposed by Park and Jung [32], which explicitly incorporates the number of questionnaire items and the expected inter-item correlation, making it particularly suitable for risk assessment surveys.

The minimum required sample size was calculated using:


$$\text{N}=\frac{Z^{\text{2}}C^{\text{2}}}{kD^{\text{2}}}(\text{1}+(k-\text{1})\rho )$$ (1)

where

Z denotes the standard normal variate corresponding to the selected confidence level,

C represents the coefficient of variation,

k is the number of questionnaire items,

D is the acceptable margin of error, and

*ρ* is the average inter-item correlation.

For this study, a five-point Likert scale was used for all questionnaire items. According to Park and Jung [32] when Likert-type scales are used, the coefficient of variation is usually less than 1.0 (C < 1). To ensure a sufficient and conservative sample size for survey-based research, a conservative value of C = 0.5 was adopted. Additionally, a conservative proportion of p = 0.5 was selected, as this is recommended for achieving acceptable sampling precision in typical survey studies [32].

The target population comprised approximately 27,000 registered engineers in the Kurdistan Region of Iraq. Using a 95% confidence level (Z=1.96), an allowable margin of error of D=0.05, k=63 questionnaire items, and a conservative inter-item correlation of ρ=0.5, the minimum required sample size was calculated as n=195. The final number of valid responses exceeded this threshold, ensuring sufficient statistical robustness for the analysis.

The final datasets comprised 378 valid responses, substantially exceeding the minimum required sample size of 195. This sample size satisfies established recommendations for robust multivariate and rank-based statistical analyses, ensuring stable and reliable results [33,34]. In addition, sample sizes greater than 300 are widely considered sufficient for achieving stable factor loading in structural validation techniques such as factor analysis [31]. Accordingly, the obtained sample is considered statistically adequate for the analyses conducted in this study.

### Reliability test

To evaluate the internal consistency of the multi-item survey instrument used in this study, reliability analysis was conducted using Cronbach’s alpha in IBM SPSS Statistics. Cronbach’s alpha is a well-established statistical coefficient used to evaluate the extent to which items within a scale consistently measure the same underlying construct and is widely applied in construction management research before further statistical analysis [35–37]. In construction research, Cronbach’s alpha is widely used to verify the reliability of questionnaire data prior to substantive analysis. The interpretation of Cronbach’s alpha commonly follows established threshold values: coefficients below 0.60 indicate poor reliability, values between 0.60 and 0.69 suggest marginal reliability, values from 0.70 to 0.79 indicate acceptable reliability, coefficients between 0.80 and 0.89 reflect good internal consistency, and values of 0.90 or above indicate excellent reliability, representing highly stable and consistent measurement of respondents perceptions [38–40].

### Risk matrix formulation

The risk assessment analysis can be conducted using various methods. The Risk Matrix method was selected as the primary analytical tool because the collected data consists of ordinal variables derived from Likert scales (1–5). Both types of risk matrices can be used to evaluate risk factors. The 2D risk matrix attempts to translate emotional responses into rational thought processes, simplifying a multi-dimensional problem into a two-dimensional format. This approach often leads to a superficial and restrictive assessment of risk [17].

On the other hand, risk management workshops are more effective when using the 3D risk matrix [22,41]. It makes it easier to examine and evaluate risks in a three-dimensional setting, enabling users to position them, visualize their contributions, and assess their comprehension. Additionally, by allowing facilitators to concentrate on particular regions of the risk cube, this tool speeds up talks and enhances the qualitative and quantitative methods of detecting and evaluating significant risks [41]. The 3D risk model helps with budget planning for risk treatment by considering the time value of risk [22]. Incorporating an extra dimension into the standard two-dimensional risk matrix to consider the resources assigned for risk management (the budget size) leads to the development of a three-dimensional coordinate system [25].

A 2D matrix can calculate the risk score by multiplying the probability by the impact, as follows [5,16]:


R=P*I (2)

Where

R = Risk Score

P = Probability

I = Impact.

**Fig 2:**
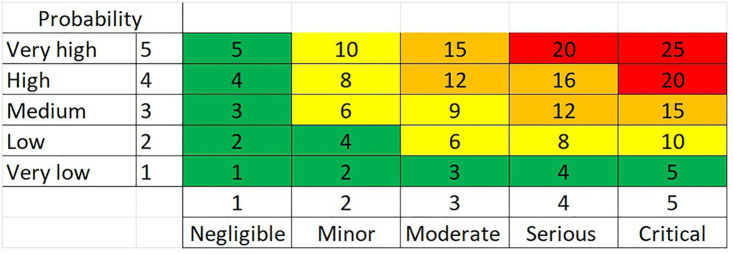
(2D) Risk matrix.

The 2D risk matrix is used to determine the status of risks. A standard 5x5 matrix classifies risks into five types: Very Low, Low, Medium, High, and Very High [42,43], as shown in Fig 2.

Based on Pritchard and PMP [44]the risk score can be determined by knowing the probability and impact. The impact may refer to cost, schedule, or other measurable factors, which may also be a combination or multiple scales for each parameter.

In the 3D risk matrix, the risk score can be calculated by multiplying the three data points as follows [41]:


R=P* (Ic * It) (3)

Risk scores can be calculated by multiplying the probability by the impact combination.


R = P * (Ic + It) (4)

Where

R = Risk Score

P = Probability

Ic = Cost Impact

It = Time Impact

**Fig 3:**
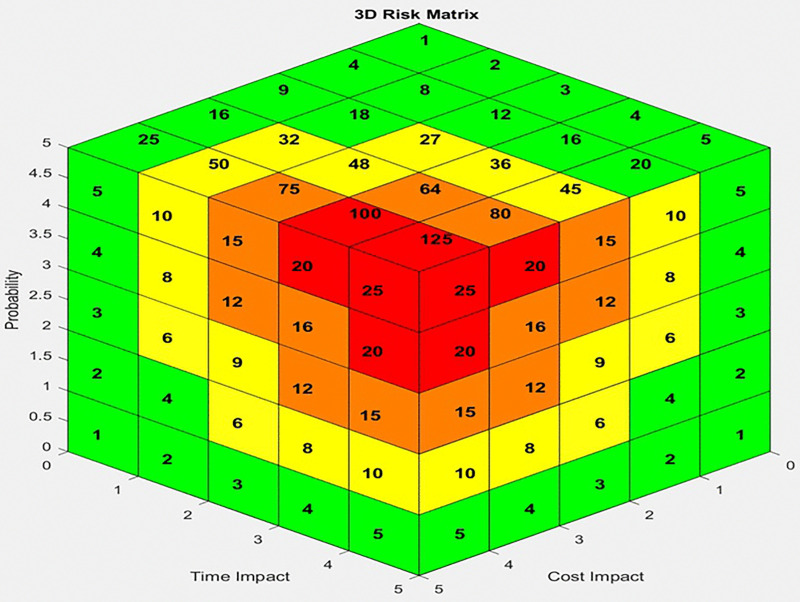
Multiplicative (3D) risk matrix.

**Fig 4:**
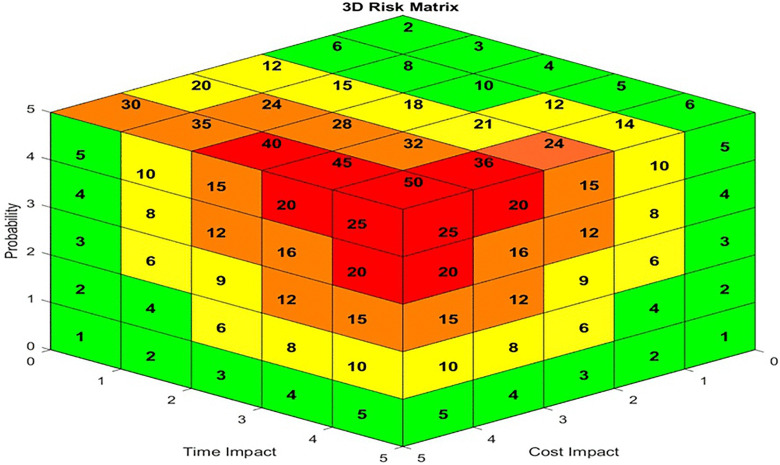
Additive (3D) Risk Matrix.

Based on equations 3 and 4, a 3D matrix can be developed to display the risk scores calculated using each formula. The additive 3D risk matrix corresponding to Equation 3 is shown in Fig 3, while the multiplicative matrix is shown in Fig 4. The coloring is done according to Lane and Hrudey [45], with each surface colored independently. Each color can be explained as mentioned in Table 1 [46]:

**Table 1:** Risk matrix color legends.

Color	Risk Level	Decision
Green	Low	Acceptable
Yellow	Medium	Acceptable after review
Orange	High	Acceptable but requires increased control and monitoring
(Red)	Critical	Unacceptable

Validation and statistical justification are critical for determining which of the developed risk matrices exhibits superior sensitivity. Accurate risk ranking is essential for enabling project managers to prioritize and address critical risk factors effectively. To this end, this study employs Spearman Rank Correlation to empirically assess the proposed 3D models. Correlation is a relationship measure among different parties or factors, and the strength and direction of the relationship [47]. The validation process first compares the rankings derived from traditional separate 2D analyses (Probability-Cost and Probability-Time) against those generated by the 3D Additive and Multiplicative matrices. Subsequently, for robust justification, the researchers established a benchmark ranking based on Probability x Maximum Impact (P x max (Ic, It)). This benchmark was compared against the 3D Additive and Multiplicative models to identify which framework best captures the true criticality of risk factors.

## Results and discussions

### Respondent profile

**Fig 5:**
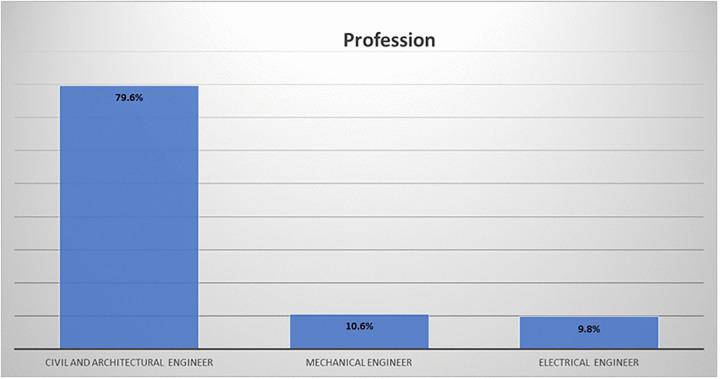
Respondent profession.

The study employed a dual RBS-WBS framework to structurally evaluate construction risks. Empirical data were gathered via a questionnaire form targeting the engineers that working in construction projects. A total of 378 engineers from various professions responded, as shown in Fig 5. As detailed in Table 2, the demographic analysis reveals a highly experienced cohort, with 63.5% of participants possessing more than 10 years of experience in the construction industry. This high level of seniority is significant for the study's analytical synthesis; it ensures that the subjective assessments of risk probability and impact are grounded in seasoned professional judgment rather than theoretical estimation, thereby strengthening the reliability of the calculated risk scores.

**Table 2:** Respondent experience.

How long have you worked in the construction industry?				
	Frequency	Percent	Valid Percent	Cumulative Percent
1-5 years	78	20.6	20.6	20.6
6-10 years	60	15.9	15.9	36.5
11-15 years	117	31.0	31.0	67.5
16-20 years	54	14.3	14.3	81.7
21-25 years	30	7.9	7.9	89.7
more than 25 years	39	10.3	10.3	100.0
Total	378	100.0	100.0	

The internal consistency of the data that were collected for this study was analyzed using the Cronbach's alpha method; the statistical results of the reliability were summarized in Table 3. Excellent reliability was noted across all the professional groups, with an alpha value for civil engineers of 0.985, for electrical engineers of 0.979, and for mechanical engineers of 0.977, divided by the recommended value limit of 0.70. These findings suggest a high degree of consistency between the evaluation of probability, time impact, and cost impact by respondents on the risk factors assessed. The consistency in the application of different engineering disciplines provides a higher degree of confidence in the robustness of the measurement approach and contributes when the risk scores are used in other analyses.

**Table 3:** Reliability statistics result.

Case Processing Summary				
What is your profession?			N	%
Civil, Engineer	Cases	Valid	301	80%
		Excluded^a^	0	0.0
Electrical Engineer	Cases	Valid	37	10%
		Excluded^a^	0	0.0
Mechanical Engineer	Cases	Valid	40	11%
		Excluded^a^	0	0.0
		Total	378	100%
**Reliability Statistics**				
What is your profession?	Cronbach's Alpha	N of Items		
Civil, Architectural, Surveyor Engineer	0.985	108		
Electrical Engineer	0.979	75		
Mechanical Engineer	0.977	75		

### Results of risk analysis

The collected data were analyzed using three distinct risk assessment approaches: the conventional two-dimensional (2D) risk matrix and two alternative three-dimensional (3D) risk matrix formulations. The 2D matrix evaluates risks separately based on probability and either cost or time impact, whereas the 3D matrices integrate probability with both cost and time impacts to provide a more comprehensive prioritization of risks. To facilitate interpretation, the calculated risk scores were classified into severity levels (e.g., Low, Moderate, High) using the coloring thresholds and decision criteria defined in Table 1, allowing for a visual comparison of how each model differentiates critical risks.

The risk factors were ranked in descending order based on their calculated risk scores (where Rank 1 represents the highest criticality). In instances where multiple risk factors yielded identical scores, the risk with the higher aggregate impact rating was prioritized and ranked higher. If both the risk score and the impact ratings were identical, the risks were assigned the same rank.

### Earthwork risks

For civil works, fourteen risk factors associated with earthwork activities were evaluated by the respondents, and the results are summarized in Table 4. The calculated risk scores indicate that all identified earthwork risks fall within the medium-risk category when assessed individually, suggesting that these risks are generally manageable but require continuous monitoring during project execution.

**Table 4:** Risk analysis related to Earthwork.

				2D Matrix (Cost Impact)			2D Matrix (Time Impact)		Multiplicative (3D) Risk Matrix		Additive (3D) Risk Matrix	
Risk Code	Risk Factor	P	I_c_	R = P*I_c_	Rank	I_t_	R = P*I_t_	Rank	R = P* (I_c_* I_t_)	Rank	R = P*(I_c_ + I_t_)	Rank
A1.1.	Design changes,	3.00	3.31	9.94	7	3.69	11.07	3	36.68	3	21.01	6
A1.2.	Unclear scope of work,	3.06	3.20	9.79	8	3.34	10.23	8	32.73	10	20.02	9
A1.3.	Low productivity of equipment,	2.93	3.25	9.51	10	3.45	10.12	11	32.84	9	19.63	10
A1.4.	Unavailability of equipment,	3.06	3.31	10.13	6	3.63	11.09	2	36.73	2	21.22	5
A1.5.	Equipment breakdown,	3.14	3.28	10.31	4	3.52	11.05	4	36.26	4	21.36	4
A1.6.	Delay in supplying materials,	3.08	3.17	9.77	9	3.49	10.75	6	34.09	8	20.52	7
A1.7.	Poor quality materials,	3.39	3.36	11.40	1	3.15	10.69	7	35.89	5	22.08	2
A1.8.	Delay of Laboratory results about materials approvals& tests,	3.00	3.07	9.22	13	3.40	10.22	9	31.38	11	19.44	11
A1.9.	Unexpected inclement weather,	3.27	3.21	10.51	3	3.39	11.10	1	35.63	6	21.61	3
A1.10.	Site obstacles (access, existing services, size of the location...etc),	2.90	2.87	8.33	14	3.12	9.04	14	25.95	14	17.37	14
A1.11.	Unforeseen site conditions,	3.07	3.08	9.44	11	3.24	9.92	12	30.55	13	19.36	12
A1.12.	Unavailability of a nearby source of appropriate filling soils,	2.89	3.23	9.34	12	3.34	9.65	13	31.18	12	18.99	13
A1.13.	Inadequate preliminary survey and site information,	3.05	3.37	10.27	5	3.35	10.21	10	34.38	7	20.48	8
A1.14.	Unrecognized soil structure/unforeseen ground condition,	3.32	3.39	11.26	2	3.32	11.04	5	37.44	1	22.30	1

Among the identified risk factors, unrecognized soil structure/unforeseen ground condition emerged as the highest-ranked risk when cost and time impacts were considered jointly. Although this risk did not consistently achieve the highest rank when cost impact and time impact were assessed separately, its combined influence resulted in the greatest overall risk score. This finding highlights the importance of considering the cumulative effect of multiple impact dimensions when prioritizing earthwork risks.

Other high-ranking risks include poor quality materials, unexpected inclement weather, and equipment-related issues, all of which demonstrate relatively high probability values coupled with moderate-to-high impact levels. These risks are likely to influence earthwork performance through delays, rework, and increased operational costs if not adequately managed.

Overall, the ranking results indicate that geotechnical uncertainty, material-related issues, and environmental conditions represent the most critical sources of risk in earthwork activities. These findings emphasize the need for early site investigation, quality control of materials, and proactive planning for adverse environmental conditions in earthwork operations.

### Concrete work risks

The risk analysis for concrete works is summarized in Table 5. The results indicate that most risks fall within the medium-risk category; however, three risks are highlighted in the high-risk (orange) zone when considering cost impact. In contrast, all risks are classified as medium risk when assessed solely based on time impact.

**Table 5:** Risk analysis related to Concrete work.

				2D Matrix (Cost Impact)			2D Matrix (Time Impact)		Multiplicative (3D) Risk Matrix		Additive (3D) Risk Matrix	
Risk Code	Risk Factor	P	I_c_	R = P*I_c_	Rank	I_t_	R = P*I_t_	Rank	R = P* (I_c_* I_t_)	Rank	R = P* (I_c_ + I_t_)	Rank
A.2.1	Lack of coordination,	3.18	3.16	10.06	9	3.41	10.86	9	34.31	9	20.92	9
A.2.2	Poor quality work,	3.44	3.50	12.06	3	3.34	11.50	5	40.32	4	23.57	4
A.2.3	Labor availability,	3.22	3.25	10.45	8	3.55	11.41	6	37.08	6	21.86	6
A.2.4	Low productivity of labour,	3.22	3.33	10.71	5	3.46	11.14	7	37.06	7	21.85	7
A.2.5	Equipment breakdown (Mixer, Pump, and crane tower)	3.52	3.35	11.79	4	3.49	12.28	3	41.18	3	24.08	3
A.2.6	Delay in supplying materials (Concrete, steel)	3.30	3.24	10.68	6	3.56	11.75	4	38.02	5	22.42	5
A.2.7	Poor quality of form works and failure during the casting of concrete,	3.59	3.58	12.87	1	3.60	12.92	1	46.32	1	25.80	1
A.2.8	Delay of Laboratory results pertaining to materials/samples approvals& tests.	3.16	3.07	9.72	10	3.41	10.77	10	33.10	10	20.49	10
A.2.9	Unexpected inclement weather (Rain, wind, etc.)	3.27	3.23	10.56	7	3.37	11.00	8	35.53	8	21.56	8
A.2.10	Difficulty during concrete pouring	3.07	3.01	9.23	11	3.23	9.91	11	29.80	11	19.14	11
A.2.11	Fail the test result of concrete	3.44	3.63	12.49	2	3.58	12.33	2	44.76	2	24.81	2

Across all ranking methods, poor quality of form work and failure during concrete casting consistently emerged as the highest-ranked risk. This risk achieved the highest scores across cost, time, and combined impact formulations, indicating its critical influence on project performance. Similarly, failure of concrete test results and equipment breakdowns (mixers, pumps, and tower cranes) were repeatedly ranked among the top risks, demonstrating strong agreement across the different assessment approaches.

These results suggest that, although the absolute risk scores differ between methods, the relative prioritization of critical risks remains largely consistent for concrete works.

### Finishing work risks

Table 6 presents the results of the risk analysis related to finishing works. The highest-ranked risk based on combined impact is damaged materials, which ranked second when considering time impact alone. In contrast, rework was ranked as the most significant risk based on time impact, while ranking third based on cost impact.

**Table 6:** Risk analysis related to finishing work.

				2D Matrix (Cost Impact)			2D Matrix (Time Impact)		Multiplicative (3D) Risk Matrix		Additive (3D) Risk Matrix	
Risk Code	Risk Factor	P	I_c_	R = P*I_c_	Rank	I_t_	R = P*I_t_	Rank	R = P* (I_c_* I_t_)	Rank	R = P* (I_c_ + I_t_)	Rank
A.3.1	Lack of coordination,	3.26	3.30	10.76	7	3.49	11.39	7	37.58	7	22.15	7
A.3.2	Low productivity of labour	3.13	3.28	10.27	10	3.52	11.04	11	36.21	10	21.32	9
A.2.3	Materials shortage	3.16	3.22	10.18	11	3.47	10.96	12	35.28	12	21.14	12
A.2.4	Shortage of equipment	3.09	3.27	10.11	12	3.60	11.15	10	36.45	9	21.26	11
A.2.5	Effect of (quality assurance/quality control) procedures	3.34	3.28	10.94	5	3.34	11.16	9	36.55	8	22.10	8
A.2.6	Labor shortages	3.12	3.18	9.93	14	3.58	11.17	8	35.56	11	21.10	13
A.2.7	Rework	3.29	3.63	11.94	3	3.71	12.22	1	44.33	2	24.16	2
A.2.8	Materials Damaged goods	3.41	3.66	12.50	1	3.55	12.12	2	44.40	1	24.62	1
A.2.9	Improper tools	3.22	3.29	10.59	9	3.32	10.71	13	35.18	13	21.29	10
A.2.10	Material delivery	3.32	3.20	10.62	8	3.57	11.83	5	37.89	6	22.45	6
A.2.11	Material quality	3.51	3.50	12.30	2	3.35	11.76	6	41.22	3	24.06	3
A.3.12	Weather Freezing, rain or Heat	3.17	3.14	9.94	13	3.29	10.42	14	32.73	14	20.36	14
A.3.13	Working at Heights	3.50	3.28	11.46	4	3.38	11.83	4	38.76	4	23.29	4
A.3.14	Non-conforming material	3.37	3.23	10.89	6	3.52	11.86	3	38.35	5	22.75	5

This pattern illustrates the value of the 3D risk matrix: it captures multi-dimensional risk interactions that single-dimension assessments might overlook. For example, while rework is the top time-related risk, its cost impact is lower, so it does not dominate the combined ranking. Similarly, material quality, though not the highest in cost or time individually, emerges as a priority when both dimensions are considered jointly. Overall, the analysis demonstrates that integrating cost and time impacts provides a more nuanced prioritization of finishing work risks, allowing project managers to identify risks that require proactive mitigation rather than focusing solely on single-dimension severity.

### Mechanical, electrical, and plumbing (MEP) work risks

In the questionnaire addressing mechanical and electrical aspects, the same risk factors were utilized. However, the engineers requested a separate assessment for each discipline due to differences in execution, coordination requirements, and technical complexity. Plumbing-related risks were incorporated within the mechanical services assessment because of their close integration in terms of installation sequence, shared resources, and coordination interfaces. The results of both assessments are presented in Tables 7 and 8.

**Table 7:** Results of the risk analysis for mechanical work.

				2D Matrix (Cost Impact)			2D Matrix (Time Impact)		Multiplicative (3D) Risk Matrix		Additive (3D) Risk Matrix	
Risk Code	Risk Factor	P	I_c_	R = P*I_c_	Rank	I_t_	R = P*I_t_	Rank	R = P* (I_c_* I_t_)	Rank	R = P* (I_c_ + I_t_)	Rank
B.1.1	Design changes,	3.30	3.55	11.72	8	3.98	13.12	4	46.57	3	24.83	6
B.1.2	Incomplete designs	3.53	3.60	12.69	4	3.90	13.75	2	49.49	2	26.44	2
B.1.3	Late issue of drawings and documents	3.18	3.15	10.00	18	3.68	11.67	11	36.75	14	21.67	13
B.1.4	Unclear scope of work,	3.28	3.18	10.40	16	3.55	11.63	12	36.91	12	22.02	12
B.1.5	Lack of coordination,	3.75	3.25	12.19	6	3.53	13.22	3	42.96	7	25.41	4
B.1.6	Excessive inspections and audits	3.18	3.35	10.64	15	3.23	10.24	22	34.30	19	20.88	18
B.1.7	Poor quality work	3.65	3.60	13.14	2	3.50	12.78	5	45.99	4	25.92	3
B.1.8	Labor availability	3.10	3.13	9.69	20	3.35	10.39	20	32.45	22	20.07	22
B.1.9	Low productivity of labour	2.95	3.23	9.51	21	3.73	10.99	16	35.44	15	20.50	20
B.1.10	Delay in supplying materials	3.18	3.38	10.72	14	3.75	11.91	9	40.18	9	22.62	11
B.1.11	Poor quality materials	3.45	3.63	12.51	5	3.48	11.99	8	43.46	6	24.50	8
B.1.12	Unavailability of materials	3.25	3.43	11.13	9	3.55	11.54	14	39.52	10	22.67	10
B.1.13	Non-conforming material	3.10	2.98	9.22	22	3.73	11.55	13	34.35	18	20.77	19
B.1.14	Un predicted technical problems during construction	3.10	3.33	10.31	17	3.58	11.08	15	36.85	13	21.39	15
B.1.15	Unexpected inclement weather	3.00	2.88	8.63	23	3.45	10.35	21	29.76	23	18.98	23
B.1.16	Site obstacles (access, existing services, size of the location, etc)	2.88	2.78	7.98	25	3.05	8.77	25	24.33	25	16.75	25
B.1.17	Late involvement of mechanical services engineers.	3.38	3.25	10.97	10	3.48	11.73	10	38.12	11	22.70	9
B.1.18	Non-involvement of specialist designers	3.58	3.65	13.05	3	3.45	12.33	7	45.02	5	25.38	5
B.1.19	Poor coordination of design inputs	3.58	3.40	12.16	7	3.48	12.42	6	42.24	8	24.58	7
B.1.20	Services conflicting with the structural frame	3.28	3.28	10.73	12	3.25	10.64	18	34.86	16	21.37	16
B.1.21	Inappropriate procurement path for Mechanical.	3.15	3.13	9.84	19	3.38	10.63	19	33.22	21	20.48	21
B.1.22	Inexperience Specialist contractors	3.93	3.80	14.92	1	3.85	15.11	1	57.42	1	30.03	1
B.1.23	Differences in the location of equipment as designed & as installed	3.20	3.35	10.72	13	3.18	10.16	23	34.04	20	20.88	17
B.1.24	Difficulty in inspection, commissioning and maintenance.	3.38	3.23	10.88	11	3.18	10.72	17	34.56	17	21.60	14
B.1.25	Difficulties in the identification of access points and services	3.00	2.78	8.33	24	2.98	8.93	24	24.77	24	17.25	24

**Table 8:** Results of risk analysis for electrical work.

				2D Matrix (Cost Impact)			2D Matrix (Time Impact)		Multiplicative (3D) Risk Matrix		Additive (3D) Risk Matrix	
Risk Code	Risk Factor	P	I_c_	R = P*I_c_	Rank	I_t_	R = P*I_t_	Rank	R = P* (I_c_* I_t_)	Rank	R = P* (I_c_ + I_t_)	Rank
C.1.1	Design changes,	3.03	3.62	10.96	7	3.59	10.88	8	39.41	4	21.84	8
C.1.2	Incomplete designs	3.38	3.27	11.05	6	3.49	11.78	3	38.52	6	22.83	5
C.1.3	Late issue of drawings and documents	3.08	3.35	10.33	9	3.76	11.57	5	38.79	5	21.90	7
C.1.4	Unclear scope of work,	3.14	3.14	9.83	14	3.32	10.42	13	32.68	14	20.25	14
C.1.5	Lack of coordination,	3.43	3.41	11.69	5	3.43	11.78	2	40.12	3	23.47	3
C.1.6	Excessive inspections and audits	2.68	3.05	8.17	24	3.27	8.75	24	26.72	23	16.92	24
C.1.7	Poor quality work	3.65	3.51	12.82	3	2.92	10.65	12	37.42	8	23.47	4
C.1.8	Labor availability	3.08	2.86	8.83	22	3.00	9.24	20	26.48	24	18.07	21
C.1.9	Low Productivity of labour	2.97	3.00	8.92	20	3.35	9.96	16	29.89	18	18.88	18
C.1.10	Delay in supplying materials	3.19	3.19	10.17	10	3.59	11.46	6	36.56	9	21.63	10
C.1.11	Poor quality materials	3.70	3.68	13.61	1	3.14	11.61	4	42.67	2	25.22	2
C.1.12	Unavailability of materials	2.86	3.32	9.52	17	3.76	10.76	9	35.78	11	20.29	13
C.1.13	Non-conforming material	2.97	3.14	9.32	19	3.59	10.69	11	33.50	13	20.01	15
C.1.14	Unpredicted technical problems during construction	3.05	3.27	9.99	12	3.41	10.40	14	34.01	12	20.39	11
C.1.15	Unexpected inclement weather	2.68	2.81	7.52	25	3.30	8.82	23	24.80	25	16.34	25
C.1.16	Site obstacles (access, existing services, size of the location, etc)	2.89	2.89	8.36	23	3.32	9.61	18	27.80	20	17.98	22
C.1.17	Late involvement of electrical services engineers.	3.19	3.16	10.08	11	3.22	10.26	15	32.43	15	20.34	12
C.1.18	Non-involvement of specialist designers	3.46	3.41	11.78	4	3.19	11.03	7	37.57	7	22.81	6
C.1.19	Poor coordination of design inputs	3.27	3.35	10.96	8	3.27	10.69	10	35.84	10	21.65	9
C.1.20	Services conflicting with the structural frame	2.92	3.24	9.47	18	3.03	8.84	22	28.66	19	18.30	20
C.1.21	Inappropriate procurement path for electrical.	3.08	3.16	9.74	16	2.84	8.74	25	27.65	21	18.49	19
C.1.22	Inexperience Specialist contractors	3.73	3.65	13.61	2	3.38	12.60	1	45.97	1	26.21	1
C.1.23	Differences in the location of equipment as designed & as installed	3.14	3.14	9.83	13	3.16	9.91	17	31.08	16	19.74	16
C.1.24	Difficulty in inspection, commissioning and maintenance.	3.08	3.19	9.83	15	3.05	9.41	19	30.01	17	19.24	17
C.1.25	Difficulties in the identification of access points and services	2.92	3.03	8.84	21	3.08	8.99	21	27.22	22	17.83	23

In relation to mechanical work, twenty-five risk factors were evaluated and ranked using both 2D and 3D risk matrix approaches. Inexperience of specialist contractors was ranked the highest across all methods, indicating a consistently high level of risk. Incomplete design was ranked second based on the combined risk impact formulation, highlighting the strong influence of design-related issues on both cost and time performance. These results indicate that risks associated with design quality and specialist expertise in mechanical works achieve high combined risk scores and therefore require careful control to mitigate their impact on project outcomes.

For electrical works, the results show that electrical systems represent a critical component of construction projects, with risks that can substantially affect both cost and schedule performance. The analysis identifies poor-quality materials, inexperienced specialist contractors, and poor quality of work as the most significant cost-related risks. Although most electrical risks are generally classified within the medium-risk category in terms of time impact, several risks attain higher combined risk scores when cost and time impacts are considered together.

### 3D model validation

The validation analysis utilized Spearman’s Rank Correlation to assess the sensitivity of the proposed 3D risk matrices against the established benchmark, defined as probability multiplying maximum impact (P * max (Ic, It)). The statistical results, presented in Table 9, indicate that while both 3D models demonstrate high validity (p < .001), the 3D Additive model exhibits superior alignment with the benchmark, evidenced by a higher correlation coefficient of 0.901 compared to the 3D Multiplicative model, which is equal to 0.893. This suggests that the Additive framework more accurately captures the true criticality of risk factors as defined by the benchmark. Furthermore, the exceptionally strong correlation observed between the Additive and Multiplicative rankings themselves confirms a high degree of internal consistency across both proposed methodologies, though the Additive model remains the preferred choice for prioritizing critical risks due to its closer proximity to the benchmark ranking.

**Table 9:** Correlation result.

Correlations					
			Rank Multiplicative	Rank Additive	Rank of Score Max Impact
Spearman's rho	Rank Multiplicative	Correlation Coefficient	1.000	.967^**^	.893^**^
		Sig. (2-tailed)	.	.000	.000
		N	89	89	89
	Rank Additive	Correlation Coefficient	.967^**^	1.000	.901^**^
		Sig. (2-tailed)	.000	.	.000
		N	89	89	89
	Rank of Score Max.Impact	Correlation Coefficient	.893^**^	.901^**^	1.000
		Sig. (2-tailed)	.000	.000	.
		N	89	89	89

**. Correlation is significant at the 0.01 level (2-tailed).

## Conclusions

Risk management is a crucial aspect of project management. In construction projects, risk factors can negatively impact project goals. The risk impacts vary, but this study focuses on cost and time impacts for different risk factors in various construction projects. To define risk factors, the study combined the WBS and RBS. A 5-point Likert scale questionnaire was designed to quantify these risk factors in terms of probability, cost impact, and time impact. In total, 378 engineers with different experiences and professions responded. The study also developed two distinct 3D risk matrices: the first was based on multiplying all three data points, while the second focused on multiplying probability by the combination of cost and time impact. All risk factors were ranked based on scores derived from the 2D traditional method and both 3D matrices. To determine which model was superior, the study employed Spearman Rank Correlation to evaluate sensitivity against a benchmark defined as Probability multiplied by Maximum Impact (P × max(Ic, It)). The results confirm that while both 3D models are statistically valid, the Combinatorial (additive) model exhibits superior alignment with the benchmark higher correlation coefficient of 0.901 compared to the multiplicative model, 0.893. This statistical evidence confirms that the Combinatorial framework captures the true criticality of risks more accurately than simple multiplication. Based on the combination of a 3D risk matrix, it can be concluded that “unrecognized soil structure/unforeseen ground condition” poses the highest risk for earthwork packages. The critical risk associated with concrete works is the “poor quality of form works and failure during the casting of concrete,” which scored a 25.8. In the finishing work package, “damaged goods” is the top-ranked risk factor, with a score of 24.6. For mechanical and electrical work packages, the risk factor related to “inexperienced specialist contractors” is considered the highest risk.

The findings provide clear implications for research and practice. Practically, the 3D risk matrix enables project managers to identify critical risk factors based on their combined cost and time impacts, supporting more effective prioritization and resource allocation. From a research perspective, this study demonstrates the value of multi-dimensional risk assessment frameworks and provides a validated methodology that can be extended to other project types or integrated with quantitative performance data.

The study is limited by its focus on the Kurdistan Region and reliance on subjective expert judgment. Future research should replicate the methodology in other geographic contexts, incorporate objective historical project data, and expand the framework to include additional impact dimensions, such as safety or environmental considerations.
