# Correction: Critical risk identification in construction projects using 2D and 3D risk matrices

**DOI:** 10.1371/journal.pone.0347505

**Published:** 2026-04-16

**Authors:** Shamal Ali Othman, Dalshad Kakasor Ismael Jaff, Ahmet Öztaş

In Fig 1, there are errors related to the arrangement and classification of risk factors within the integrated Work Breakdown Structure (WBS) and Risk Breakdown Structure (RBS) framework. Please see the correct Fig 1 here.

**Fig 1:**
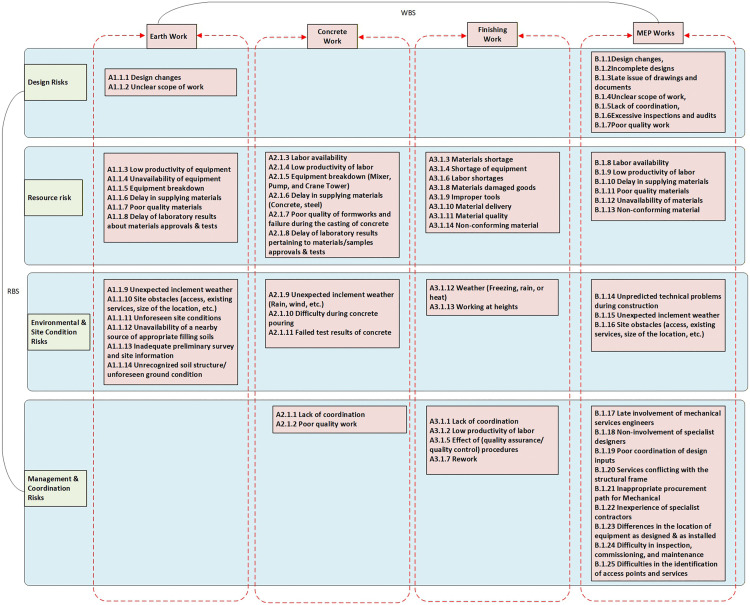
RBS and WBS Integration.
